# Investigation of phytoplankton community structure and formation mechanism: a case study of Lake Longhu in Jinjiang

**DOI:** 10.3389/fmicb.2023.1267299

**Published:** 2023-10-05

**Authors:** Yongcan Jiang, Yi Wang, Zekai Huang, Bin Zheng, Yu Wen, Guanglong Liu

**Affiliations:** ^1^PowerChina Huadong Engineering Corporation Ltd., Hangzhou, Zhejiang Province, China; ^2^College of Environmental and Resource Sciences, Zhejiang University, Hangzhou, Zhejiang, China; ^3^College of Resources and Environment, Huazhong Agricultural University, Wuhan, Hubei, China; ^4^State Key Laboratory of Environmental Criteria and Risk Assessment, Chinese Research Academy of Environmental Sciences, Beijing, China

**Keywords:** phytoplankton community, structural characteristics, spatial distance, environmental factors, formation mechanism

## Abstract

In order to explore the species composition, spatial distribution and relationship between the phytoplankton community and environmental factors in Lake Longhu, the phytoplankton community structures and environmental factors were investigated in July 2020. Clustering analysis (CA) and analysis of similarities (ANOSIM) were used to identify differences in phytoplankton community composition. Generalized additive model (GAM) and variance partitioning analysis (VPA) were further analyzed the contribution of spatial distribution and environmental factors in phytoplankton community composition. The critical environmental factors influencing phytoplankton community were identified using redundancy analysis (RDA). The results showed that a total of 68 species of phytoplankton were found in 7 phyla in Lake Longhu. Phytoplankton density ranged from 4.43 × 10^5^ to 2.89 × 10^6^ ind./L, with the average density of 2.56 × 10^6^ ind./L; the biomass ranged from 0.58–71.28 mg/L, with the average biomass of 29.38 mg/L. *Chlorophyta*, *Bacillariophyta* and *Cyanophyta* contributed more to the total density, while *Chlorophyta* and *Cryptophyta* contributed more to the total biomass. The CA and ANOSIM analysis indicated that there were obvious differences in the spatial distribution of phytoplankton communities. The GAM and VPA analysis demonstrated that the phytoplankton community had obvious distance attenuation effect, and environmental factors had spatial autocorrelation phenomenon, which significantly affected the phytoplankton community construction. There were significant distance attenuation effects and spatial autocorrelation of environmental factors that together drove the composition and distribution of phytoplankton community structure. In addition, pH, water temperature, nitrate nitrogen, nitrite nitrogen and chemical oxygen demand were the main environmental factors affecting the composition of phytoplankton species in Lake Longhu.

## 1. Introduction

Global warming exacerbates the process of lake eutrophication, which further changes the distribution, phenology, abundance, composition, and trophic interactions of phytoplankton (Yan et al., 2017; Ajani et al., 2020). Phytoplankton is the important primary producer of aquatic ecosystems and plays a key role in the process of material circulation and energy flow (Lin, 2023). The complexity of their community structure in terms of temporal and spatial distribution is the premise to maintain the functional integrity of the ecosystem (Rodríguez-Gómez et al., 2022). As one of the essential indicators of aquatic environment, the structure, abundance and seasonal succession of phytoplankton communities are highly susceptible to environmental factors, and its sensitivity to hydrological environment can effectively indicate the health status of aquatic ecosystems and changes in water quality (Dong et al., 2021; Xu Q. S. et al., 2022; Xu Y. P. et al., 2022). Studies have confirmed that abiotic factors such as availability of light, temperature, inorganic nutrients (nitrogen, phosphate, silicates, and iron), hydrological connectivity, and biotic factors (e.g., zooplankton) can affect the growth and community succession of phytoplankton (Zhang et al., 2018; Han et al., 2021). In addition, the decomposition of phytoplankton has been shown to generate greenhouse gases (CO_2_, CH_4_, N_2_O) and release dissolved nutrients that can retroactively favor climate change and eutrophication (Li and Li, 2018). The optical feedback effect of chlorophyll in phytoplankton also leads to a warming of the lake surface, which in turn significantly affects eutrophication (Lewis et al., 1990; Park et al., 2015). Therefore, understanding the structural characteristics of phytoplankton communities and their formation mechanism is essential to strengthen the management of lake aquatic ecological environment.

Previous studies on phytoplankton communities mainly proceeded from the niche theory, connecting species, communities and environmental factors, and believed that environmental processes (environmental filtration) were the most important factors affecting the structure of phytoplankton communities, and environmental heterogeneity caused differences in species composition in different environments (Valencia et al., 2004). Nevertheless, the neutral theory based on species dispersal and stochastic processes emphasizes the impact of spatial processes on biological communities where the existence, absence, and relative abundance of species is controlled by random seeding, dispersal, ecological drift, and extinction processes (Pos et al., 2019; He et al., 2022). Several recent studies indicate that the neutral theory might play an important role in phytoplankton communities (Mutshinda et al., 2016), as it underpins unexplained (random) variation in the relative abundances of species in phytoplankton communities, or on clumpy distributions of species traits such as cell size (Vergnon et al., 2009; Burson et al., 2019). There is also a view that the small phytoplankton floating on surface waters are easily dispersed passively through hydrology (Liu H. et al., 2022; Liu X. et al., 2022). This leads to highly similar algal community compositions at distant locations largely independent of environmental variables (Wu et al., 2023).

At present, numerous studies also have shown that environmental processes and spatial processes (diffusion limitation) play a role in the construction of phytoplankton communities, whereas their relative importance may vary across ecosystems and spatiotemporal scales (Heino et al., 2015; Chang et al., 2023). Compared with the ocean, the phytoplankton community composition of lake systems is more easily affected by spatial processes (Shurin et al., 2009), yet for lake phytoplankton, environmental factors (such as temperature, light, and hydrodynamic conditions) are still the main factors affecting the community structure (Wang et al., 2011; Mucina et al., 2016; Anceschi et al., 2019). Many studies have indeed confirmed that non-biological environmental variables (such as macronutrients and micronutrients), acting as indispensable protein cofactors and nutritional elements, were the main driving factors for abundance and composition of phytoplankton in aquatic ecosystems (Chiellini et al., 2020; Xue et al., 2021). Particularly, changes in nitrogen and phosphorus loads may lead to phytoplankton niche differentiation, and this different response to nutrient availability is mainly attributed to differences in explanatory degrees of niche differentiation and neutral competition (Burson et al., 2019). Consequently, it is of great significance to explore the phytoplankton community structure in lakes and its response mechanism to environmental factors and spatial processes for exploring the status of aquatic ecosystems.

In this study, the phytoplankton of Lake Longhu was the research object. Based on the monitoring of the spatial distribution of phytoplankton and water quality, the spatial differences in the community structure were analyzed. And the principal coordinate analysis of the neighbor matrix was used to identify the spatial structure of phytoplankton community formation, combined with variance decomposition to quantify the role of environmental factors, spatial processes, and their joint actions in the phytoplankton communities of Lake Longhu, and finally use canonical correspondence analysis to influence the phytoplankton community structure. It can identify significant environmental factors and provide basic data support for the water environment management and aquatic ecosystem protection of Lake Longhu.

## 2. Materials and methods

### 2.1. Study area

**Figure 1:**
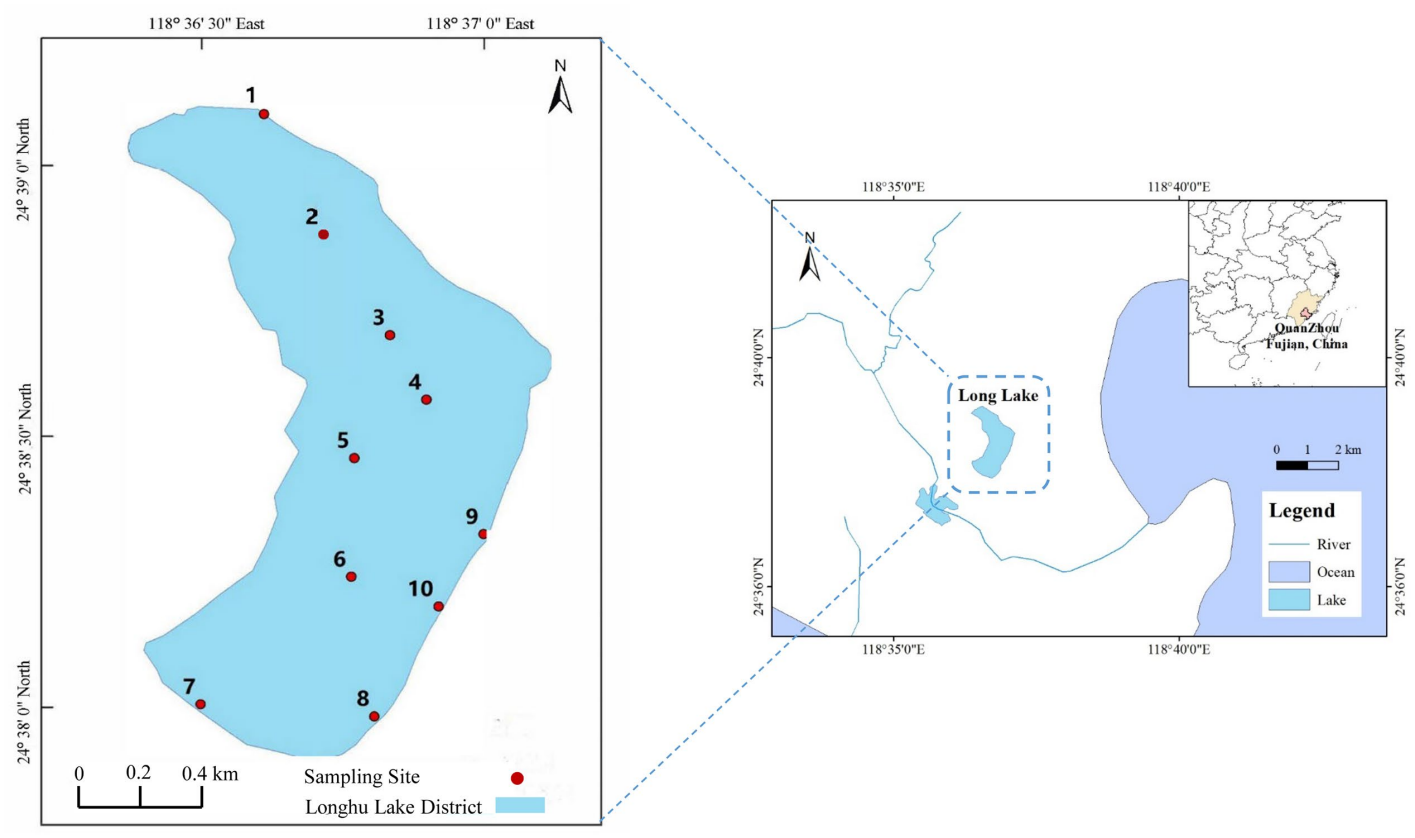
Location of Lake Longhu and the distribution of sampling sites.

Lake Longhu (24°37′53″-24°39′7”N, 118°36′22″-118°37′7″E) is the largest natural shallow lake in Fujian Province, China, with a surface area of 1.60 km^2^, a rainfall catchment area of 11.37 km^2^, and an average water depth of 2.00–3.00 m (Figure 1). Lake Longhu is also an important water source for the water supply project of Jinjiang four towns and Jinmen county, undertaking the 4.40 m^3^s^−1^ distribution of water in Jinji Gate Reservoir of Jinjiang. The total reservoir capacity of Lake Longhu is 4.05 million m^3^, the annual runoff is 5.15 million m^3^, and the water exchange cycle of the entire lake area is about 10 days. With a certain capacity for regulation and storage, and self-regulating through the overflow outlet in the lower reaches of the lake. The climate of this area belongs to the subtropical monsoon climate, the average annual rainfall is 911–1,231 mm. The rainfall is mainly concentrated in the typhoon and rainstorm season in July and September, accounting for about 70% of the total rainfall (Wang and Ye, 2009).

### 2.2. Sample collection and preparation

According to the topography and surrounding environment of the lake, ten sampling sites were set up in Lake Longhu (as shown in Figure 1), and the longitude and latitude information of each sampling site was detailed in Supplementary Table S1. Sampling site 1 is in the entrance of Lake Longhu, sampling sites 2–7 are the open water in the lake, and sampling sites 8–10 are a small wetland in the shoreline zone of the lake. A total of 30 samples were collected from June to July in 2020. Water samples from the surface (at 0.5 m below the lake surface), middle layer (at water depth of l.5 m) and bottom layer (0.5 m on the surface of the sediment) were collected with a Patalas water collector, and the three layers of water samples were mixed. The mixed water sample were treated with nitric acid (pH < 2) and filtered with 0.45 μm microporous membrane, and then stored at 4°C for subsequent analysis.

The collection of phytoplankton in the field was divided into qualitative sampling and quantitative sampling. The qualitative samples were fished out of surface water with No. 25 phytoplankton nets, fixed with Lugol’s reagent on site and then brought back to the laboratory for identification. Quantitative samples were collected with a water collector of 1.0 L water samples at each sampling site, also fixed with 1.5% Lugol’s reagent on site, brought back to the laboratory to stand for 24–36 h, concentrated to 30 mL, and then 0.1 mL of the samples were microscographed with a counting frame to record phytoplankton species and quantities. Phytoplankton species were identified according to Hu and Wei (2006). Cell density was measured with a counting chamber under microscopic magnification of × 200–400. Algal biovolumes were calculated from cell numbers and cell size measurements. Conversion to biomass assumes that 1 mm^3^ of volume is equivalent to 1 mg of fresh weight biomass.

### 2.3. Physicochemical parameters analysis

Water temperature (WT), pH, dissolved oxygen (DO) and conductivity (EC) were determined *in situ* by the multiparameter water quality monitor (EXO, Yellow Springs Instruments, United States). Transparency of the mixed water was measured using a Cypriot disc (SD). The concentrations of total nitrogen (TN), total phosphorus (TP), ammonia nitrogen (NH_4_^+^-N), nitrate nitrogen (NO_3_^−^-N), and nitrite nitrogen (NO_2_^−^-N) in lake water were measured following standard methods (State Environmental Protection Administration of China, 2002). The chemical oxygen demand (COD_Mn_) in water sample was analyzed by potassium dichromate. Chlorophyll-a (Chla) was extracted using ethanol at 4°C. The Chla concentration was expressed as the difference in absorbance between 665 nm and 649 nm using ethanol as the control (Shi et al., 2015).

### 2.4. Statistical analysis

Calculation of Shannon-Wiener diversity index of phytoplankton communities based on species abundance. Clustering analysis was used to identify the spatial distribution of phytoplankton communities, and the Bray–Curtis similarity coefficient between sampling sites was calculated after the phytoplankton density data was converted to the fourth power root and normalized, so as to reduce the influence of excessive density of dominant species, and then hierarchical clustering was carried out by group average. Similarity Profiles (SIMPROF) analysis was used to verify whether the community structure obtained by clustering is significantly different from the random spatial structure (*p* < 0.05). One-way ANOSIM was used to verify whether there were significant differences in species composition at different sampling sites. And diversity index calculations, cluster analysis, SIMPROF analysis and ANOSIM analysis were all completed by PRIMER 6.0 (Clarke et al., 2006).

The generalized additive model (GAM) was used to fit the relationship between community heterogeneity, geographical distance and environmental distance, respectively. The community phase heterogeneity matrix was based on phytoplankton density data, and the Bray-Curtis phase dissimilarity matrix was calculated by quadruple root transformation. The geographic distance matrix was obtained by calculating the geographic distance of each pair of stations based on the latitude and longitude coordinates of the sampling sites. And the Euclidian distance matrix was calculated from the environmental variables of the sampling sites. The Bray-Curtis heterogeneity matrix and Euclidian distance matrix were completed using the “vegan” package of the R language (4.03); The geographic distance matrix was done using the package “geosphere”; GAM was done using the package “mgcv” (Team, R.C, 2014).

Principal coordinate analysis of neighborhood matrix (PCNM) was used to identify the spatial structure formed by phytoplankton communities. Variance partitioning analysis (VPA) was used to assess the extent to which environmental and spatial variables explained the variation in phytoplankton community. Before the VPA analysis, in order to ensure the simplicity of the model, the significant (*p* < 0.05) environment factors and spatial variables were selected and screened out based on the previous term, and then brought into the model for VPA analysis. The correlation between phytoplankton communities and environmental factors was analyzed by redundancy analysis (RDA). Significant environmental factors were screened out using forward selection, and model significance was detected by Monte Carlo displacement tests (499 times). PCNM vector acquisition, VPA analysis, and RDA analysis were all done using Canoco 5 software (Šmilauer and Lepš, 2003).

## 3. Results

### 3.1. Water quality of each sampling site

**Figure 2:**
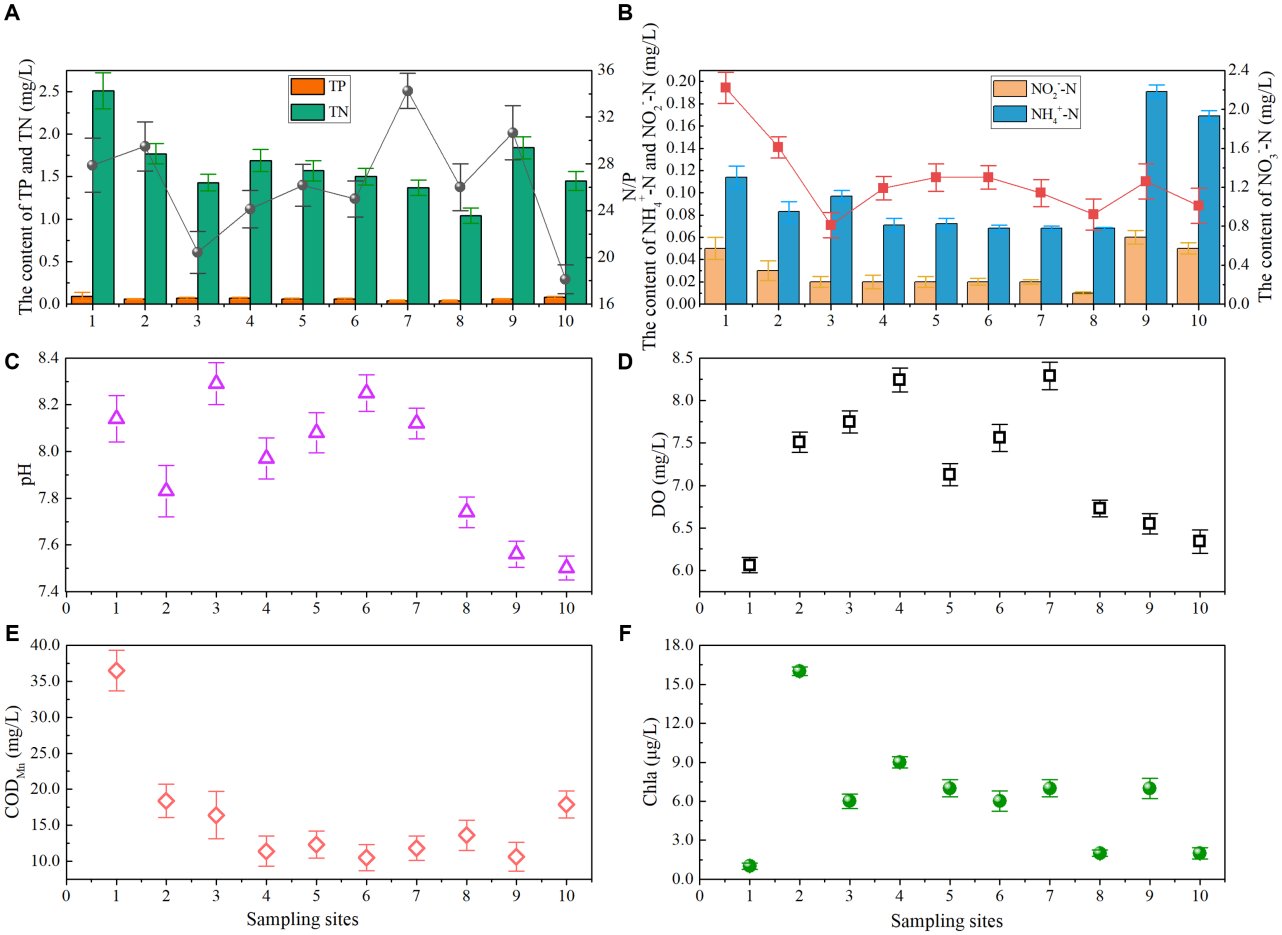
Spatial distribution of water quality parameters in Lake Longhu [(A) TP, TN, and N/P; (B) NH_4_^+^-N, NO_3_^-^-N, and NO_2_^-^-N; (C) pH; (D) DO; (E) COD_Mn_; (F) Chla].

As shown in Figure 2, the variation of physicochemical indicators of water in Lake Longhu were presented. The range of pH was between 7.50 and 8.25. pH of the sampling site from 1 to 7 was higher than that at the 8–10 sampling sites. The range of DO in water was 6.06 to 8.29 mg/L in Lake Longhu, respectively. DO value was the lowest at the entrance of sampling site, and the value of DO at 2 to 7 sampling sites was higher. SD and DO had the same variation trend (Supplementary Table S2). EC ranged from 143.0–331.0 μS/cm, and the conductivity at site 2 in the north was 159 μS/cm, which was lower than the measured value at the entrance level, indicating that the conductivity brought by the entrance water had little effect (Supplementary Table S2). And the conductivity measured at sampling sites 8–10 was generally higher, which might be mainly due to the slow flow of water. Furthermore, the chlorophyll content of water showed a lower level in general, and the contents of Chla and SD both showed a higher trend in the north than in the south. The highest chlorophyll content was found near site 2, indicating that the water environment near it was suitable for the growth and reproduction of algae. Both Chla and SD were lower in the small wetlands of sites 8 and 10, which may be due to the predominant influence of suspended particulate matter in the lake on transparency, while the influence of algae was more limited.

Judging from the change trend of COD_Mn_ concentration in Lake Longhu, the highest COD_Mn_ concentration was 36.46 mg/L at site 1, and the other sampling sites were between 10.54 and 18.35 mg/L. The content of TP ranged from 0.04 to 0.09 mg/L and TP in open water was generally lower, with the highest content at the entrance, indicating that the content of TP in Lake Longhu was affected by certain entrance water. The overall TN concentration in the water was generally higher, ranging from 1.04 to 2.51 mg/L, and the TN concentration showed a decreasing trend from north to south, in addition, the TN concentration at the entrance site 1 was also substantially higher than the average value in the lake. The concentrations of NH_4_^+^-N, NO_3_^−^-N and NO_2_^−^-N in the water were between 0.07–0.19 mg/L, 0.81–2.22 mg/L and 0.01–0.06 mg/L, respectively. And the overall trend was similar to that of TP and TN. From the northern lake area to the southern lake area, the N, P levels showed a gradual downward trend, while there was an increase at sampling sites 8 and 10, indicating that the entrance water quality of Lake Longhu was poor and may be the main source of water pollution. In terms of the overall analysis from the north to the south of the lake, the overall water quality of Long Lake was in good condition. Moreover, the ratio of N/P combined with their concentration has great effects on algal growth, physiological response, biochemical compositions, and potentially impacted the function and community structure of aquatic ecosystems (Keck and Lepori, 2012; Huang et al., 2019; Zhao et al., 2019). Generally, the N/P molar ratio required for phytoplankton growth and physiological balance was 16: 1 (Redfield, 1960), and it was changed with phytoplankton species due to their different requirements for N and P (Ho et al., 2003; Wagner et al., 2021). In this study, the N/P molar ratio of all sampling sites was greater than 16, indicating phosphorus limitation. In addition, lower phosphorus content also inhibited organic synthesis of nitrogen (Meunier et al., 2017).

### 3.2. Phytoplankton community structures

**Figure 3:**
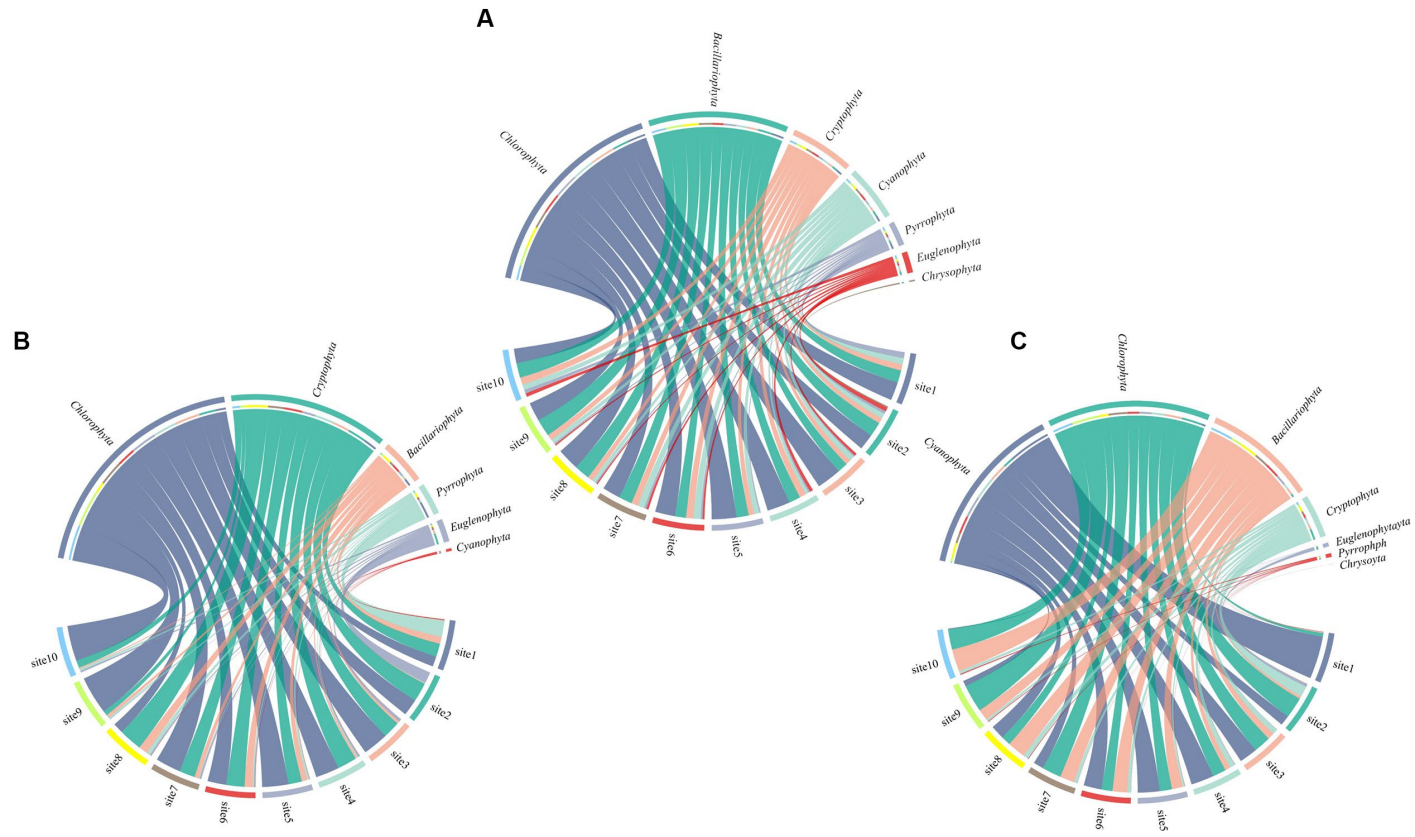
Spatial variations of phytoplankton composition percentages [**(A)** species; **(B)** biomass; **(C)** density].

**Figure 4:**
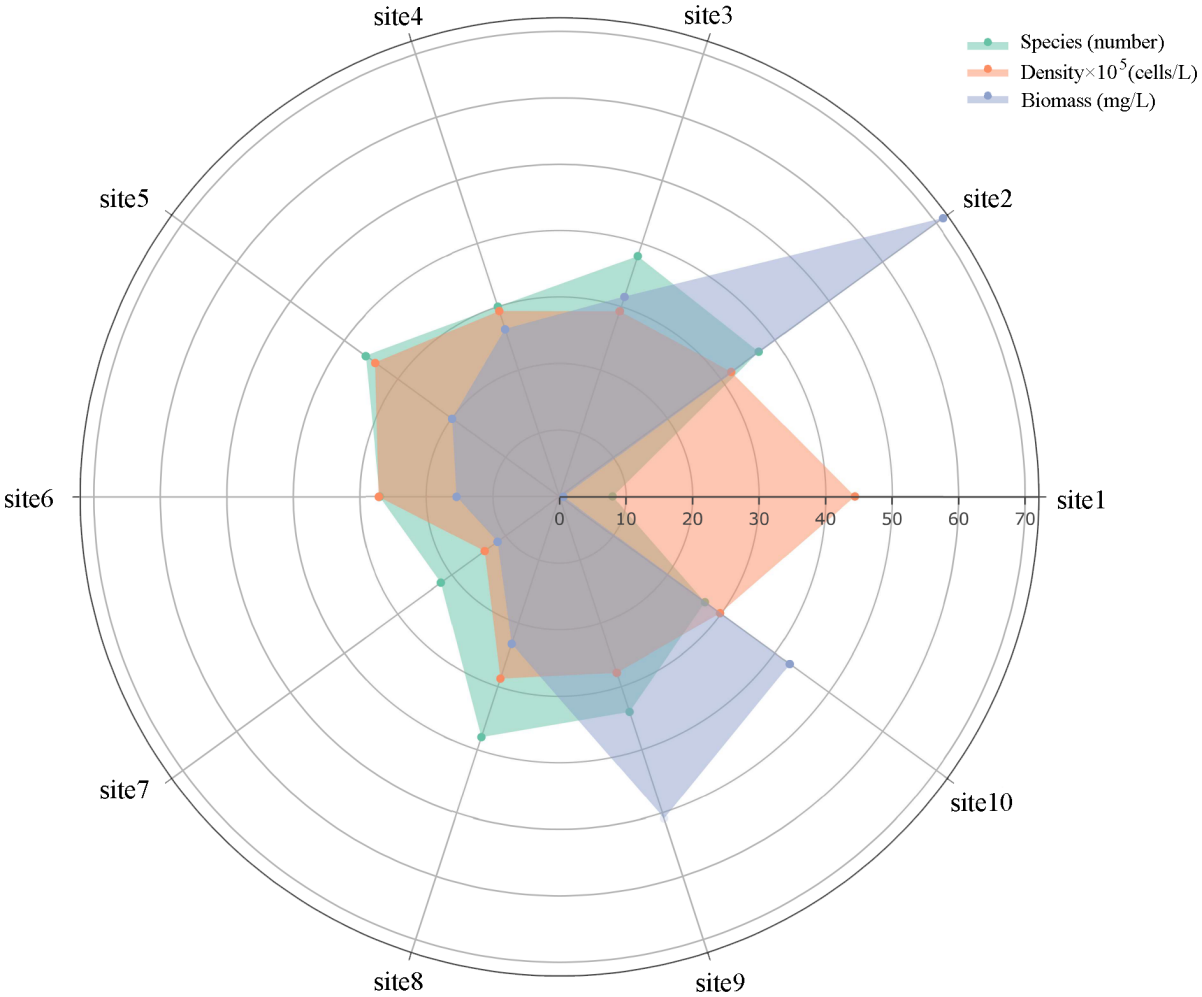
Spatial variations of phytoplankton community characteristics.

A total of 68 species of phytoplankton were identified at the sampling sites (Supplementary Table S3), *Cyanophyta*, *Chlorophyta*, and *Bacillariophyta* were identified as dominant phyla, with 29 species of *Chlorophyta*, accounting for 42.6% of the total number of species, followed by 21 species of *Bacillariophyta* (30.9%), and 7 species of *Cyanophyta*, accounting for 10.3%. *Cryptophyta*, *Euglenophyta*, *Pyrrophyta*, and *Chrysophyta* were 4, 3, 3 and 1 respectively, accounting for 5.9%, 4.4%, 4.4%, and 1.5% of the total species. The spatial variations of phytoplankton composition percentages was shown in Figure 3, and the spatial change of phytoplankton community characteristics was shown in Figure 4. The number of phytoplankton species varies from 8 to 38 species at different sampling sites, with an average number of 30 species. The least number of phytoplankton species was found at the entrance of Lake Longhu, and the general trend from the south to the north of the lake was a decrease in species.

The total density of phytoplankton was 2.56 × 10^7^ ind./L, and the density variation range was 4.43 × 10^5^–2.89 × 10^6^ ind./L, and the average density was 2.56 × 10^6^ ind./L. The total biomass of phytoplankton was 293.82 mg/L, the biomass variation ranged from 0.58 to 71.28 mg/L, and the average biomass was 29.38 mg/L. In terms of density composition, *Chlorophyta* (8.84 × 10^6^ ind./L), *Cyanophyta* (7.72 × 10^6^ ind./L), *Bacillariophyta* (4.86 × 10^6^ ind./L), and *Cryptophyta* (2.46 × 10^6^ ind./L) became the main phytoplankton of Lake Longhu in this study with abundance ratios of 34.5%, 30.1%, 19.0%, and 9.58%, respectively. With respect to biomass composition, *Chlorophyta* (154.67 mg/L) and *Cryptophyta* (88.66 mg/L) accounted for 47.95% and 31.67% of the total biomass, which were the phytoplankton with the highest proportion of biomass in Lake Longhu, followed by *Bacillariophyta* and *Pyrrophyta*, with a biomass proportion of 8.98% and 6.29%, respectively. From the overall perspective of the lake, the density and biomass of phytoplankton at the entrance were lower, and the density and biomass of phytoplankton in the open waters of the lake showed a consistent trend, while the biomass decreased from north to south, and increased at sampling sites 8, 9, and 10. Judging from the survey results, the phytoplankton in the water of Lake Longhu were mainly *Chlorophyta*, *Cyanophyta*, *Bacillariophyta* and *Cryptophyta* (Xu Y. P. et al., 2022). Further identification of algal species revealed that the dominant species of *Chlorophyta* were *Pandorina morum (Muell.) Bory* and *Chlorella* sp., the dominant species of *Cyanophyta* were *Merismopedia tenuissima Lemm.* and *Aphanizomenon* sp., and the dominant species of *Bacillariophyta* were *Cyclotella meneghiniana Kütz.* and *Melosira* sp.

### 3.3. Phytoplankton diversity index and similarity analysis

**Figure 5:**
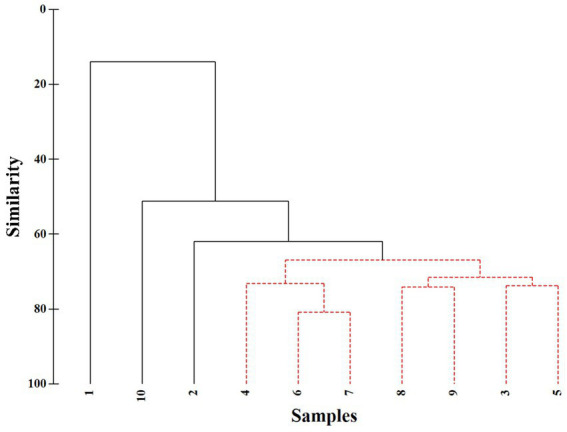
Cluster analyses for the phytoplankton community structure in Lake Longhu.

The Shannon-Wiener diversity index of the phytoplankton communities was calculated based on species abundance, and the results showed that the Shannon-Wiener diversity index of phytoplankton at different sampling sites in Lake Longhu varied from 0.47 to 2.62, with an average diversity index of 2.09 (Supplementary Figure S1), indicating that the phytoplankton community parameters at different sampling sites fluctuated widely, and there were spatial differences in the species distribution of phytoplankton. In addition, the results of cluster analysis and SIMPROF analysis also showed that there were significant differences in the spatial distribution of phytoplankton communities among sampling sites (Figure 5). At the 15% similarity level, there was a significant difference between sampling site 1 and other sampling sites (*p* = 0.001); at the 50% similarity level, there was a significant difference between sampling site 10 and other sampling sites (*p =* 0.001); and there was a significant difference between sampling site 2 and other sampling sites at the 60% similarity level (*p* = 0.005). The ANOSIM analysis showed that there were significant differences in phytoplankton community structure at different sampling sites (R^2^ = 0.933, *p* = 0.008), indicating significant spatial variability in phytoplankton community structure.

### 3.4. Distance attenuation effect of phytoplankton

**Figure 6:**
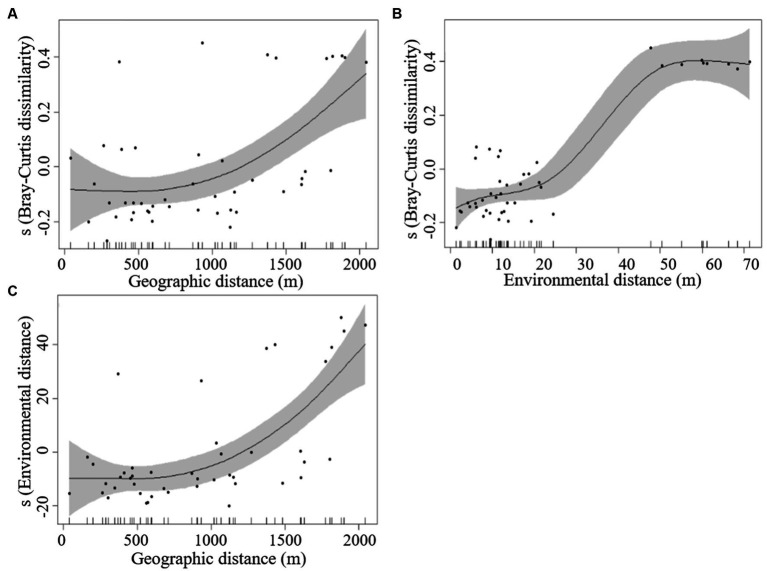
The fitted curves between Bray–Curtis dissimilarity of phytoplankton, geographic distance, and environmental distance based on GAM models [(A) Bray–Curtis dissimilarity and geographic distance; (B) Bray–Curtis dissimilarity and environmental distance; (C) environmental distance and geographic distance].

In order to further investigate the variation of phytoplankton along different geographical distances and environmental factors, a generalized additive model (GAM) was constructed with the relative values of phytoplankton abundance as response values and geographical distance and environmental distance factors as independent variables, and the fitting results of the composition GAM model showed that there was a significant distance attenuation effect in phytoplankton communities, namely the heterogeneity of community composition increased with the increase of geographical distance (R^2^ = 0.332, *p* < 0.001; Figure 6A). Environmental factors play an important role in phytoplankton community construction, and community heterogeneity increased with increasing environmental distance (R^2^ = 0.852, *p* < 0.001), while the change in community heterogeneity slowed down when the environmental distance was large enough (Figure 6B). On the other hand, there was spatial autocorrelation in environmental factors, and environmental distance increased with the increase of geographical distance (R^2^ = 0.475, *p* < 0.001; Figure 6C), which indicated that the environmental distance and water correlation factors of Lake Longhu jointly drove the composition and distribution of phytoplankton community structure.

### 3.5. Correlation of community composition and water quality parameters

The spatial structure characteristics of phytoplankton community formation were identified based on the principal coordinate analysis of the neighborhood matrix (PCNM), and the results showed that the previous selection screened out five environmental factors (pH, water temperature, NO_2_^−^-N, NO_3_^−^-N and COD_Mn_) and one spatial factor (PCNM1) had a significant impact on the phytoplankton community structure (*p* < 0.05). In addition, the variance decomposition (VPA) results showed that the environment variable (*p* = 0.002) and the spatial variable (*p* = 0.026) jointly explain 41.6% of the community variation of the phytoplankton community, respectively. The environment variable explained more community variation than the spatial variable, with the two together explaining 7.3% of the community variation (Supplementary Figure S2).

**Figure 7:**
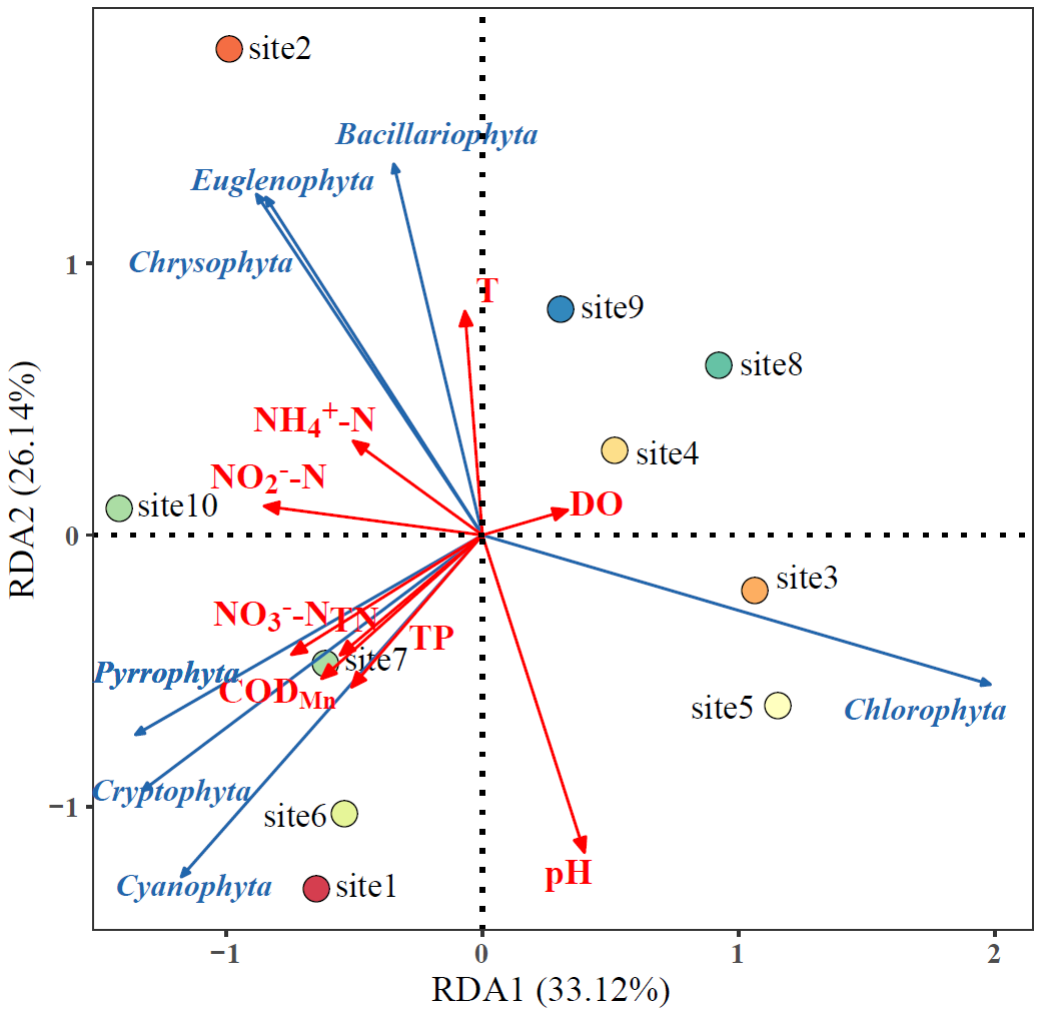
Redundancy analysis (RDA) of phytoplankton community species composition and environmental factors.

The relationship between the major drivers and phytoplankton community composition was examined based on the RDA models. The RDA explained about 59.26% of the variation in phytoplankton community composition in Lake Longhu. Most of this variation is explained by the first axis (33.12%) with the second axis explaining an additional 26.14% (Figure 7). It was clearly from the figure that pH, temperature, NO_2_^−^-N, NO_3_^−^-N and COD_Mn_ were the main environmental factors affecting the distribution of the phytoplankton community. In addition, a good correlations among NO_2_^−^-N, NO_3_^−^-N, NH_4_^+^-N, COD_Mn_, TN and TP was also demonstrated from the smaller pinch angles between environmental factors. From the effects of environmental factors on the abundance of specific phytoplankton, DO and pH mainly affected the growth of *Chlorophyta*, while water temperature, NO_2_^−^-N and NH_4_^+^-N mainly affected the relative abundance of *Chrysophyta*, *Euglenophyta* and *Bacillariophyta*, and the growth of *Pyrrophyta*, *Cryptophyta*, *Cyanophyta* was mainly related to the amount of NO_2_^−^-N, NO_3_^−^-N, NH_4_^+^-N, COD_Mn_, TN and TP.

## 4. Discussion

### 4.1. Distribution characteristics of phytoplankton communities and their influencing factors

Overall, the species, density and biomass of phytoplankton showed that the sampling site 1 at the entrance was the smallest, sampling site 2 was the most suitable for algae growth, and phytoplankton trends decreased at sampling sites 2 to 7, while phytoplankton growth at sampling sites 8, 9, and 10 showed a slight increase trend, indicating that the phytoplankton species and biomass at the entrance were the least and the structure was relatively simple, while the water of the vast lake surface was suitable for the growth and reproduction of phytoplankton. However, in general, the composition of the phytoplankton structure in the lake was relatively simple, the stability was good, and the overall water quality was also better. Phytoplankton are extremely sensitive and perceptive to lake water quality. It has been shown that different species of phytoplankton are often found together in water with frequent blooms, dominate in different areas of the water, and show dominant population succession in time series (Zhang et al., 2016). Since realized niches of phytoplankton in various environmental factors are different and also contribute to explaining the succession of phytoplankton community in the complicated environment under the multiple stresses of anthropogenic activities and climate changes. The dominant population composition reflected the pollution status of the lakes and reservoirs to a certain extent, and in terms of community composition, *Chlorophyta*, *Cyanophyta*, *Bacillariophyta* and *Cryptophyta* in Lake Longhu were the dominant populations in phytoplankton in our study. According to the density composition, the proportion of *Cyanophyta* in the entrance was the largest, and *Cyanophyta* were the most typical dominant group of eutrophic water, which indicated that there was a certain degree of pollution in the entrance water. Hydraulic residence time or flow rate can also affect the occurrence of *Cyanophyta*. Some studies have confirmed that the slower flow rate and longer retention time of lake and reservoir water intercepted higher nutrients, which was conducive to the growth and propagation of *Cyanophyta* (Han et al., 2022). Studies have also demonstrated that *Cyanophyta* as prokaryotes have a significant competitive advantage over other eukaryotic producers (such as *Chlorophyta*, *Bacillariophyta*, etc.) under nutrient-rich conditions (Paerl et al., 2011). Furthermore, temperature as a climatic factor also significantly influenced the growth of *Cyanophyta*, and relatively higher temperature enhanced the competitiveness of *Cyanophyta* with other eukaryotic phytoplankton (Kim et al., 2020). It has been noted that some *Cyanophyta* are recorded to have a dominant position in comparison with diatom above 25°C (Berg and Sutula, 2015).

With the gradual decrease of *Cyanophyta* density from the north to the south of Lake Longhu, the dominant algae population of the water changed to *Chlorophyta*, which also indicated that the probability of eutrophication in the lake gradually decreased. There are many factors that cause this change, the most important of which is nutrient elements. Nitrogen and phosphorus are the main drivers of phytoplankton growth in lakes, and it has been found that *Chlorophyta* have a stronger growth advantage than *Cyanophyta* and grow faster compared to *Cyanophyta* under conditions of sufficient phosphate concentration (Klisarova et al., 2023). In addition, the nitrate nitrogen content in Lake Longhu is higher (Figure 2), and some researchers have found that *Cyanophyta* are less competitive in this environment (Chen et al., 2017), which is consistent with the results of this study. In addition, *Chlorophyta*, as important primary producers in aquatic and terrestrial ecosystems, exhibit great diversity in morphological and ultrastructural characteristics, which leads to their better viability and wider ecological niche compared to other phytoplankton (Fields et al., 2021). Interestingly, *Cryptophyta* were more sensitive to temperature than nutrients (Rao et al., 2021). Furthermore, *Cryptophyta* have a unique auxiliary pigment for photosynthesis and a competitive advantage in lake with their own advantages as a dominant taxon in July when light is stronger and water temperature is higher, so *Cryptophyta* has higher biomass in the vast lake surface (Ding et al., 2018; Cheng et al., 2019; Tian et al., 2022).

As one of the most diverse and abundant classes of photosynthetic microalgae, diatoms are known to play an indispensable role in regulating the biogeochemical cycle of nutrients such as carbon, nitrogen and silica in lake waters (Liu et al., 2012). As the genus with the highest proportion of diatom populations, *Bacillariophyta* has strong viability due to its special carbon concentration and fixation mechanism. Studies have shown that *Bacillariophyta* are the dominant population among phytoplankton in rivers, which have physiological competitive advantages over the growth of other phytoplankton in rivers, while the growth of *Bacillariophyta* is significantly correlated with phosphorus, and the TP concentration in Lake Longhu is 0.04–0.09 mg/L, which is conducive to the growth of *Bacillariophyta* (Vello et al., 2023). In addition, Beaver et al. (2013) found that the biomass of *Bacillariophyta* increased with decreasing hydraulic residence time, which was the opposite of the adaptation of *Cyanophyta* to the environment (Beaver et al., 2013). Moreover, *Bacillariophyta* potentially take up nitrate to serve as a sink for electrons during periods of imbalance between light energy harvesting and utilization, and this mechanism is apparently not present in non-diatom species, thus *Bacillariophyta* growth well in RW ponds are associated with high nitrate supply (Berg et al., 2003; Liu H. et al., 2022; Liu X. et al., 2022). The dominant phytoplankton population varies in different months, and most species only become dominant species in a certain month, so in order to better understand the composition of phytoplankton populations in Lake Longhu, it is necessary to monitor and study the phytoplankton in Lake Longhu at different times. The lake as a whole in this study was in a poor-mesotrophic state. In terms of total phytoplankton biomass and density, the *Cyanophyta*, *Chlorophyta* and *Bacillariophyta* contributed more to the total density, and *Chlorophyta* and *Cryptophyta* contributed more to the total biomass, which may be caused by multiple influences such as the population characteristics of phytoplankton and environmental factors.

### 4.2. Environmental factors drive phytoplankton community succession

In lakes where eutrophication occurs frequently, different dominant populations compete with each other and undergo succession, and the mechanisms by which eutrophication occurs are always the result of the interaction of complex and environmental factors (Han et al., 2023). Phytoplankton spatial processes and environmental factors are important ecological processes for community composition and biodiversity maintenance in biological assemblages (Chase and Myers, 2011; Laland et al., 2016). The results of GAM model and VPA analysis showed that there was a significant correlation between phytoplankton community variability and geographical distance and environmental factors (Figure 6; Supplementary Figure S2). Both environmental and spatial factors significantly explained the variation of phytoplankton community, indicating that spatial processes and environmental factors play equally important roles in the construction of phytoplankton community in Lake Longhu, which breaks the conclusion that phytoplankton community structure is mainly affected by environmental processes in previous studies (Xu Q. S. et al., 2022; Xu Y. P. et al., 2022; Shuwang et al., 2023).

Further analysis of specific environmental impact factors is of great significance for exploring the formation mechanism of phytoplankton community structure and the development direction of community succession. Many previous studies have focused on the environmental influences of phytoplankton in specific lakes and reservoirs. Yang et al. (2021) found that TN, conductivity, water temperature and altitude factors affected the density of phytoplankton and the distribution of dominant species in reservoirs in the arid region of northwest China (Yang et al., 2021). Wang et al. (2022) found that water temperature, pH, COD_Mn_ and total nitrogen were the main driving factors affecting the seasonal variation of phytoplankton community structure and density in urban lakes in cold areas. And in our study, pH, water temperature, nitrite nitrogen, nitrate nitrogen and COD_Mn_ were the main drivers affecting the phytoplankton community structure in Lake Longhu (Figure 7). In addition, The number of *Gymnodinium aeruginosum Stein.* was higher at sampling site 10, its community structure was significantly different from other sampling sites by cluster analysis and SIMPROF analysis (Figure 5). The RDA analysis further showed that there was a positive correlation between phytoplankton species composition and nitrite nitrogen as well as ammonia nitrogen at sampling site 10. The monitoring results of Lake Longhu over the past 10 years showed that the eutrophication problem of Lake Longhu had a tendency to intensify year by year, with the level of nitrogen and phosphorus gradually increased (Li and Li, 2018). Furthermore, the phytoplankton survey in Lake Longhu showed that *Euglenophyta* was occasionally species, and the eutrophication process that occurred changed the species composition of the phytoplankton in Lake Longhu. The results of cluster and SIMPROF analysis showed that there were significant differences in the phytoplankton community structure between sampling site 1 and other sampling sites, and there was a positive correlation between the phytoplankton species composition and pH, TP and COD_Mn_. COD_Mn_ is a chemically measured amount of reducing substance in the water that needs to be oxidized, representing the water body in an oxidation/reduction environment. The source of COD_Mn_ is generally the organic matter in the water, and the organic matter in the water body mainly comes from the discharge of domestic sewage and industrial wastewater, as well as the influx of animals and plants after the decomposition of decay with rainfall (Liu et al., 2023). The chemical oxygen demand reflects the degree of organic pollution in water, and the larger the COD_Mn_, the more serious the organic pollution of the water (Wang et al., 2021). Sampling site 1 was located at the entrance of Lake Longhu, where the higher Mn content in the upstream water and organic pollution affected the species composition of phytoplankton.

### 4.3. Mechanisms of phytoplankton community structure formation

The results of the GAM model showed that there was a significant distance attenuation effect on phytoplankton communities, and community heterogeneity increased with geographic distance, indicating that diffusion restriction affected phytoplankton community formation. However, environmental distances also increased with geographic distances, suggesting that there was spatial autocorrelation of environmental factors in lake and the interpretation of community structure by spatial processes may be due to environmental filtering (Ma et al., 2022). The results of VPA analysis showed that spatial processes and environmental processes together explained 7.3% of variation in community structure, which was higher than purely spatial processes, and spatial processes mainly influenced the construction of phytoplankton community structure mainly through the joint action of environmental processes. The environmental variables explained the 41.6% of the variation in phytoplankton community structure in Lake Longhu, which was higher than the interpretation rate of the spatial variables, indicating that the deterministic processes filtered by the environment were the main cause of the variation in phytoplankton community structure. Studies have also shown the importance of spatial and environmental processes as community building factors in freshwater lakes (Herrera-Silveira and Morales-Ojeda, 2009; Alahuhta et al., 2023), the relative importance of which depends on the dispersal capacity of species, environmental gradients, and spatial extent (Heino et al., 2015). Increased species dispersal capacity contributes to reducing dispersal restriction and increasing the importance of environmental processes (Shurin et al., 2009). The water exchange period in the study area was short, and the environmental gradient difference was significant, which provided diffusion conditions for phytoplankton, and the spatial distribution of phytoplankton was affected by the regional cluster effect, and the role of environmental processes was obvious (Cui et al., 2016).

## 5. Conclusion

Based on the investigation of water quality and phytoplankton community composition in Lake Longhu, this study further explored the formation mechanism and influencing factors of phytoplankton using methods such as cluster analysis (CA), analysis of similarities (ANOSIM), redundancy analysis (RDA) and generalized additive model (GAM). During the investigation period, a total of 7 phytoplankton species were detected in Lake Longhu, mainly *Chlorophyta*, *Bacillariophyta*, *Cyanophyta*, accounting for 42.6%, 30.9%, and 10.3% respectively, and the proportion of other species was smaller. The results of CA and ANOSIM analysis showed that there were obvious spatial differences in phytoplankton communities. The GAM and variance partitioning analysis further showed that there was a significant distance attenuation effect in phytoplankton communities and spatial autocorrelation in environmental factors, which significantly affected the construction of phytoplankton communities. In addition, pH, water temperature, nitrite nitrogen, nitrate nitrogen and COD_Mn_ were the main environmental factors affecting the composition of phytoplankton species in Lake Longhu based on the RDA analysis.

## Data availability statement

The original contributions presented in the study are included in the article/Supplementary material, further inquiries can be directed to the corresponding author.

## Author contributions

YJ: Conceptualization, Data curation, Formal analysis, Investigation, Methodology, Project administration, Writing – original draft, Writing – review & editing. YiW: Data curation, Formal analysis, Methodology, Software, Supervision, Writing – original draft. ZH: Data curation, Formal analysis, Methodology, Software, Supervision, Writing – review & editing. BZ: Funding acquisition, Project administration, Resources, Supervision, Validation, Writing – review & editing. YuW: Funding acquisition, Project administration, Resources, Supervision, Validation, Writing – review & editing. GL: Project administration, Supervision, Validation, Visualization, Writing – review & editing.
