# Supplementary material for: Investigation of phytoplankton community structure and formation mechanism: a case study of Lake Longhu in Jinjiang
Source: Front Microbiol. 2023 Oct 5;14:1267299. doi: 10.3389/fmicb.2023.1267299 (PMC10585031; doi:10.3389/fmicb.2023.1267299)
Supplement: Supplementary file 1 [file Data_Sheet_1.docx]

**Supplementary material**

**Investigation of phytoplankton community structure and formation mechanism: A case study of Lake Longhu in Jinjiang**

Yongcan Jiang ^1,2†*^, Yi Wang ^3†^, Zekai Huang ^4^, Bin Zheng ^1^, Yu Wen ^1^, Guanglong Liu ^3^

^1^ PowerChina Huadong Engineering Corporation Ltd., Hangzhou 311122, Zhejiang Province, China

^3^ College of Environmental and Resource Sciences, Zhejiang University, HangZhou 310058, Zhejiang Province, China

^3^ College of Resources and Environment, Huazhong Agricultural University, Wuhan,, China

^4^ State Key Laboratory of Environmental Criteria and Risk Assessment, Chinese Research Academy of Environmental Sciences, Beijing, China

**E-mail：**[jiangyc6@zju.edu.cn](mailto:jiangyc6@zju.edu.cn)

^†^ These authors contributed equally to this work and share first authorship

**Table S1.** The longitude and latitude information of each sampling site in Lake Longhu.

| Sampling sites | Longitude  (°) | Latitude  (°) |
| --- | --- | --- |
| 1 | 118.611 | 24.6482 |
| 2 | 118.614 | 24.6469 |
| 3 | 118.617 | 24.6425 |
| 4 | 118.622 | 24.6410 |
| 5 | 118.618 | 24.6372 |
| 6 | 118.616 | 24.6325 |
| 7 | 118.618 | 24.6311 |
| 8 | 118.620 | 24.6334 |
| 9 | 118.620 | 24.6348 |
| 10 | 118.620 | 24.6331 |

**Table S2.** Water quality indicators at Lake Longhu sampling sites.

|  | Site 1 | Site 2 | Site 3 | Site 4 | Site 5 | Site 6 | Site 7 | Site 8 | Site 9 | Site 10 |
| --- | --- | --- | --- | --- | --- | --- | --- | --- | --- | --- |
| WT (℃) | 31.58 | 35.53 | 33.44 | 32.12 | 31.18 | 31.16 | 31.24 | 31.44 | 30.09 | 30.35 |
| SD (cm) | 36.00 | 47.00 | 33.00 | 48.00 | 36.00 | 41.00 | 35.00 | 30.00 | 28.00 | 25.00 |
| EC (µS/cm) | 243.0 | 159.0 | 143.0 | 177.0 | 206.0 | 128.0 | 195.0 | 297.0 | 331.0 | 287.0 |
| Hg (µg/L) | 0.00 | 0.70 | 0.13 | 0.17 | 0.11 | 0.24 | 0.15 | 0.00 | 0.00 | 0.00 |
| Mn (µg/L) | 78.15 | 31.26 | 34.90 | 23.85 | 22.03 | 23.81 | 14.40 | 10.61 | 29.60 | 14.60 |
| Cu (µg/L) | 3.03 | 2.78 | 3.84 | 3.68 | 3.20 | 3.22 | 3.40 | 2.68 | 3.06 | 1.73 |
| Zn (µg/L) | 52.07 | 17.94 | 17.39 | 20.76 | 33.44 | 18.86 | 20.43 | 16.03 | 20.47 | 17.06 |
| As (µg/L) | 0.94 | 0.75 | 0.89 | 0.84 | 0.86 | 0.86 | 0.85 | 0.87 | 0.76 | 0.88 |
| Cd (µg/L) | ND | ND | ND | ND | ND | ND | ND | ND | ND | ND |
| Pb (µg/L) | 1.89 | 1.08 | 1.43 | 1.19 | 1.17 | 1.13 | 0.98 | 0.85 | 1.60 | 1.10 |
| Fe (µg/L) | 98.81 | 88.58 | 205.94 | 149.37 | 120.78 | 134.93 | 134.40 | 92.70 | 177.84 | 93.62 |

**Table S3.** List of phytoplankton in Lake Longhu.

| Phylum/Species | |  | |
| --- | --- | --- | --- |
|  | ***Cyanophyta*** |  |  |
| 1 | *Aphanocapsa delicatissima* | 2 | *Merismopedia tenuissima* Lemm. |
| 3 | *Dactylococcopsis irreguloris* | 4 | *Aphanizomenon* sp. |
| 5 | *Anabaena* sp. | 6 | *Pseudoanabaena* sp. |
| 7 | *Planktothrix agardii* |  |  |
|  | ***Chlorophyta*** |  |  |
| 1 | *Scenedesmus quadricauda* | 2 | *Scenedesmus bijuga* |
| 3 | *Scenedesmus bicaudatus* | 4 | *Scenedesmus dimorphus* |
| 5 | *Scenedesmus denticulatus* | 6 | *Dictyosphaerium pulchellum* |
| 7 | *Ankistrodesmus falcatus* | 8 | *Chlorella* sp. |
| 9 | *Ankistrodesmus falcatus* | 10 | *Chlamydomonas globosa* Snow |
| 11 | *Pandorina morum* | 12 | *Ptermonas tetraptera* |
| 13 | *Eudorina elegans*. | 14 | *Pediastrum simplex var.duodenarium* |
| 15 | *Tetraedron trigonum*. | 16 | *Pediastrum duplex var.clathratum* |
| 17 | *Pediastrum tetras* | 18 | *Actinastrum hantzschii* Lagerheim |
| 19 | *Crucigenia quadrata* | 20 | *Oocystis* sp. |
| 21 | *Crucigenia apiculata* | 22 | *Schroederia setigera* Lemm. |
| 23 | *Tetrastrum glabrum* | 24 | *Schroederia nitzschioides* |
| 25 | *Selenastrum minutum* | 26 | *Chodatella wratislaviensis* |
| 27 | *Coelastrum microporum* | 28 | *Staurastrum gracile* |
| 29 | *Golenkinia radiata* |  |  |
|  | ***Bacillariophyta*** |  |  |
| 1 | *Cyclotella meneghiniana* | 2 | *Cocconeis placentula* |
| 3 | *Melosira granulata var.angustissima* | 4 | *Navicula placentula* |
| 5 | *Melosira varians* | 6 | *Navicula cryptocephala* |
| 7 | *Melosira distans* | 8 | *Navicula* spp. |
| 9 | *Synedra* sp. | 10 | *Gomphonema angustatum* |
| 11 | *Synedra berolinensis* | 12 | *Cymbella ventricosa* |
| 13 | *Synedra acus* | 14 | *Achnanthes exigua* |
| 15 | *Synedra ulna* | 16 | *Gyrosigma acuminatum* |
| 17 | *Diatoma* sp. | 18 | *Pinnularia* sp. |
| 19 | *Nitzschia paradoxa* | 20 | *Surirella* *anguatata* |
| 21 | *Nitzschia* spp. |  |  |
|  | ***Cryptophyta*** |  |  |
| 1 | *Cryptomonas ovata* | 2 | *Cryptomonas rostrata* |
| 3 | *Cryptomonas erosa* | 4 | *Chroomonas acuta* |
|  | ***Euglenophyta*** |  |  |
| 1 | *Euglena oxyuris*. | 2 | *Trachelomonas* sp. |
| 3 | *Euglena caudata* |  |  |
|  | ***Pyrroptata*** |  |  |
| 1 | *Glenodinium pulvisculus* | 2 | *Gymnodinium aeruginosum* |
| 3 | *Peridinium* sp. |  |  |
|  | ***Chrysophyta*** |  |  |
| 1 | *Chrysococsus rufescens* |  |  |


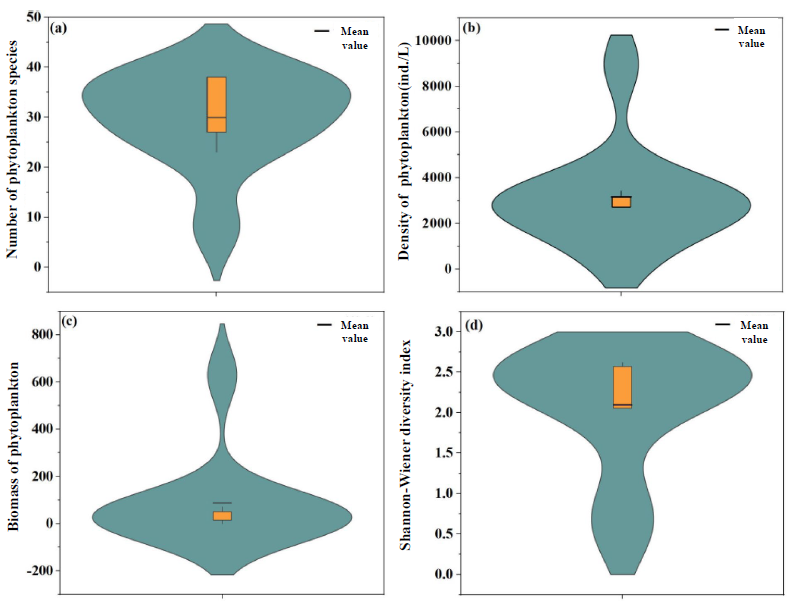


**Figure S1.** Number of phytoplankton species (a), density of phytoplankton (b), biomass of phytoplankton (c) and Shannon-Wiener (d) diversity of phytoplankton in Lake Longhu.


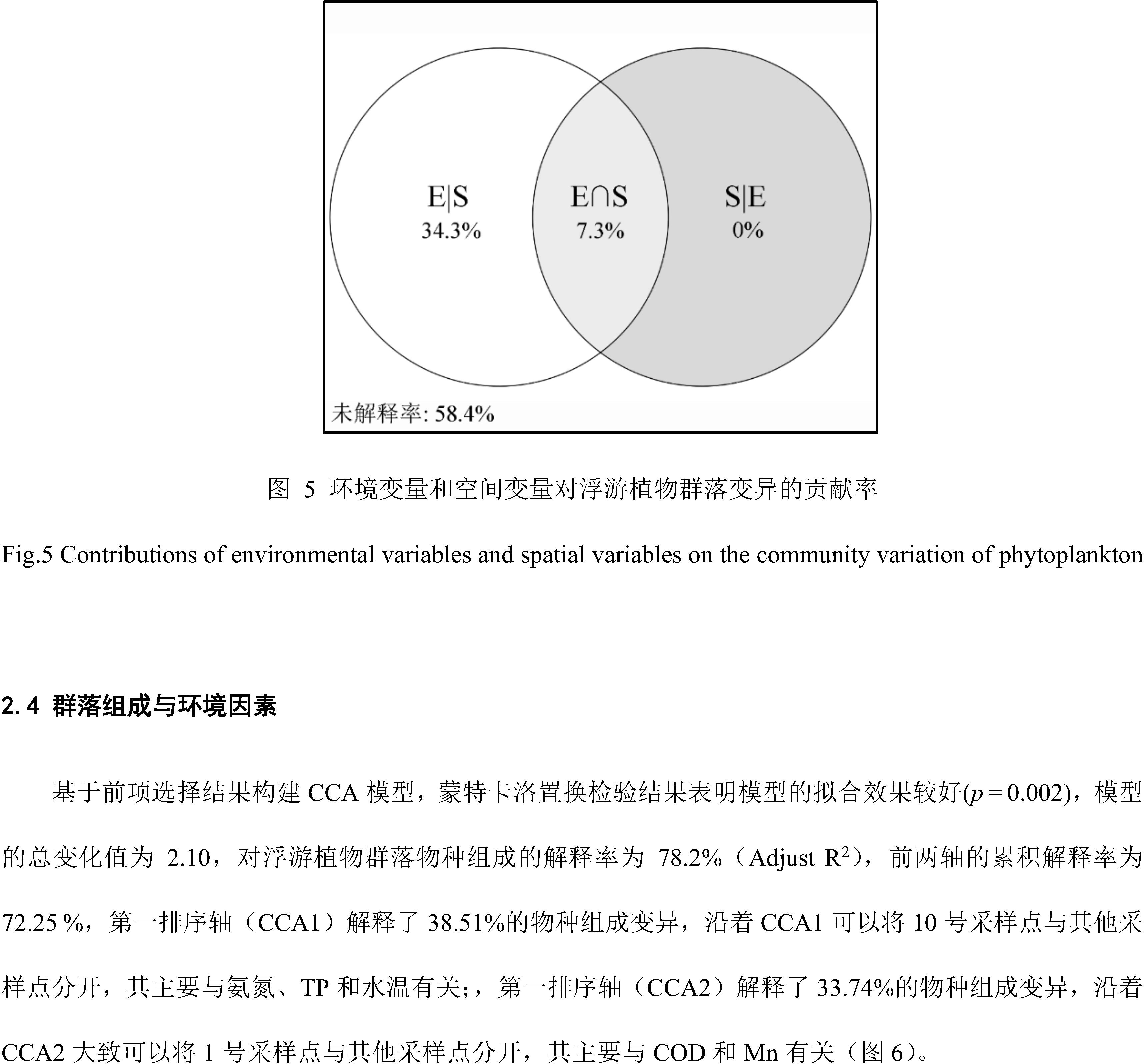


**Unexplained Rate**

**Figure S2.** Contributions of environmental variables (E) and spatial variables (S)on the community variation of phytoplankton.
